# Time-refraction optics with single cycle modulation

**DOI:** 10.1515/nanoph-2023-0126

**Published:** 2023-05-31

**Authors:** Eran Lustig, Ohad Segal, Soham Saha, Eliyahu Bordo, Sarah N. Chowdhury, Yonatan Sharabi, Avner Fleischer, Alexandra Boltasseva, Oren Cohen, Vladimir M. Shalaev, Mordechai Segev

**Affiliations:** Physics Department and Solid State Institute, Technion-Israel Institute of Technology, Haifa, Israel; School of Electrical and Computer Engineering, Birck Nanotechnology Center and Purdue Quantum Science and Engineering Institute, Purdue University, West Lafayette, IN, USA; School of Chemistry, Tel Aviv University, Tel Aviv, Israel

**Keywords:** Photonic time-crystals, time-varying media, ultrafast optics

## Abstract

We present an experimental study of optical time-refraction caused by time-interfaces as short as a single optical cycle. Specifically, we study the propagation of a probe pulse through a sample undergoing a large refractive index change induced by an intense modulator pulse. In these systems, increasing the refractive index abruptly leads to time-refraction where the spectrum of all the waves propagating in the medium is red-shifted, and subsequently blue-shifted when the refractive index relaxes back to its original value. We observe these phenomena in the single-cycle regime. Moreover, by shortening the temporal width of the modulator to ∼5–6 fs, we observe that the rise time of the red-shift associated with time-refraction is proportionally shorter. The experiments are carried out in transparent conducting oxides acting as epsilon-near-zero materials. These observations raise multiple questions on the fundamental physics occurring within such ultrashort time frames, and open the way for experimenting with photonic time-crystals, generated by periodic ultrafast changes to the refractive index, in the near future.

## 1 Introduction

Large and fast modulations of the macroscopic electromagnetic (EM) properties of materials can have far reaching consequences [1–4]. Unlike the common low-amplitude modulation or slow modulation in time, when the macroscopic response is large and fast enough (as we study here) even a single “step” modulation can result in dramatic effects [5]. However, conventional nonlinear optics does not operate in this regime. Namely, the ultrafast nonlinear response of most materials is very weak in the optical regime, hence such changes have to be induced resonantly to enhance the response [6], as common in many applications such as active mode locking [7], optical isolators [8, 9], light control [10] and applications based on nonlinear optics [1]. Alternatively, large and fast changes can occur in free plasma phase transition [11], but since such materials become heavily lossy – it is immaterial to explore the propagation of ultrafast pulses in these systems. It is therefore necessary to find some other mechanisms or materials that would enable very large changes in their EM properties at ultrafast rates. As described below, recent progress with transparent conducting oxides is now enabling experiments displaying large optically-induced response in the optical regime (see, for example, recent review Ref. [12]).

When a wave propagating in a medium experiences a sudden change in the real part of the refractive index *n*(*t*), it undergoes two fundamental process – time refraction, and time reflection [13]. In this process of temporal modulation of the EM properties of the medium, the energy carried by the propagating wave (“probe”) is not conserved, but when the medium is homogeneous the wave momentum (wave-vector) is conserved (see, e.g., Ref. [14]). That is, the time-refracted wave and the time-reflected wave have the same wave-vector as the original wave propagating in the medium. Consequently, when the refractive index is increased abruptly – the spectrum of the wave undergoes a red-shift, and when the index is decreased the spectrum undergoes a blue-shift. This spectral shift occurs in both time-refraction and time-reflection. In the optical regime, broadband frequency translation through time refraction has been recently observed in experiments [15, 16], but time-reflection was thus far demonstrated only with water waves [17] (where water propagating away from a point source was partially reflected back to the origin by changing the wave impedance in the medium) and very recently with microwaves [18]. The lack of energy conservation in such time-modulated EM media implies that the scattered wave may even be more energetic than the incident one [19], and can lead to tremendous amplification of waves by the modulation. Such time boundaries may also be used for applications such as antireflection temporal coatings [20], extreme energy transformations [21], inverse prisms [22], temporal aiming [23] and are also genuinely interesting for the fundamental physics involved [24–28]. Concatenating several time-modulations periodically can lead to the formation of wide momentum gaps, and the system acts as a photonic time-crystal (PTC) [2, 3, 29–31]. The gaps in momentum of a PTC, where the temporal frequency is complex, can lead to broadband parametric amplification, and can interact with the emission of radiation by free electrons [32], atoms [4] and classical dipoles [4]. All of these rely on the ability to induce time-interfaces: very large changes in the EM properties of materials occurring at single wave-cycle rates. In microwaves, this task is hard but reachable with present technology [18, 30]. But certainly, bringing these concepts to the optical (or near infrared) regime, where the time-interfaces can couple to electronic transitions in materials, will yield a plethora of profound novel physics ideas as well as new applications.

On this background, it is clear why materials that can change their properties at ultrafast rates have recently been attracting increasing research interest, with the quest being to find mechanisms that would display very large changes; ideally, refractive index changes on the order of unity that can occur within a few femtoseconds. Conventional nonlinear optics that relies on interaction between light and bound electrons is ultrafast, but orders of magnitude too weak for observing time-reflection and PTCs. Other mechanisms that can yield very large nonlinear response typically require transport and are therefore too slow. A great promise comes from transparent conducting oxide (TCO) materials near the epsilon near zero (ENZ) point, which were recently found to display very large light-induced refractive index changes occurring at ultrafast rates [15, 16, 33–37] (for a recent review, see Ref. [12]). In these materials, electrons can be driven within the conduction band to higher energies by ultrafast laser pulses. Since such process relies on optical excitation, which is an induced-transition, it is in principle instantaneous, and the response can be as fast as the excitation pulse. In this scheme, the excitation pulse serves as a “modulator” for other (much weaker) waves that would propagate in the medium and experience the large ultrafast time-interfaces induced by the modulator. Moreover, when the central frequency of such a weak pulse propagating within the material is near the ENZ point, the pulse experiences dramatic changes in its reflection and transmission properties. For this reason, recent experiments on time-refraction in the optical regime employed ENZ materials [15, 16, 36]. However, thus far in all of these experiments the modulating pulses ranged from tens to hundreds of femtoseconds; hence the rate of the overall change was much slower than a single cycle (a few femtoseconds) of the near infra-red probe beam used for experimenting with optical time-refraction. Under these conditions, all of those experiments did not study time-interfaces but rather the response to a gradual change in the refractive index. Interestingly, in a very recent study, fast rise times in ITOs were also estimated with a long modulator (225fs) in a clever scheme demonstrating double-slit time diffraction [38]. This study also points for the need of directly observing the fast response of ENZ materials with many of its important features [39, 40] which were not revealed thus far.

## 2 Goals

In this article, we explore the physics of time-refraction induced by a large and very short (1–2 optical cycles) modulation of the EM properties in ENZ materials. To induce significant time-refraction, we create a moving time-boundary that occurs as fast as the time-period of the propagating wave, with a refractive index change on the order of Δ*n* ∼ 0.5. Crucially, the material remains relatively transparent despite the huge relative index change, which allows us to explore new physics. The time-refraction we observe exhibits two frequency shifts induced by the index modulation. The first is a red-shift from the instantaneous rise in the refractive index. We demonstrate, for the first time, that this large refractive index change can occur within a single optical cycle. We do that by varying the duration of the modulation pulse from 30 fs down to 6 fs, and directly observe the corresponding decrease in the response time. The second frequency shift we observe is the blue-shift associated with the relaxation (decrease) of the refractive index of the material back to its original value. We observe a large blue-shift of duration similar to that of the red-shift. This implies that the relaxation process of the electrons back to their original energies (bottom of conduction band) occurs within ultrashort time scales, contrary to the expected relaxation of hot electrons in TCOs, which relies on the coupling to phonons and takes hundreds of femtoseconds. In addition to these few-fs red and blue frequency shifts, we also observe other processes, which are considerably slower, and can be deduced from the reflection and transmission coefficients of the modulated material.

## 3 Experimental setup

**Figure 1:**
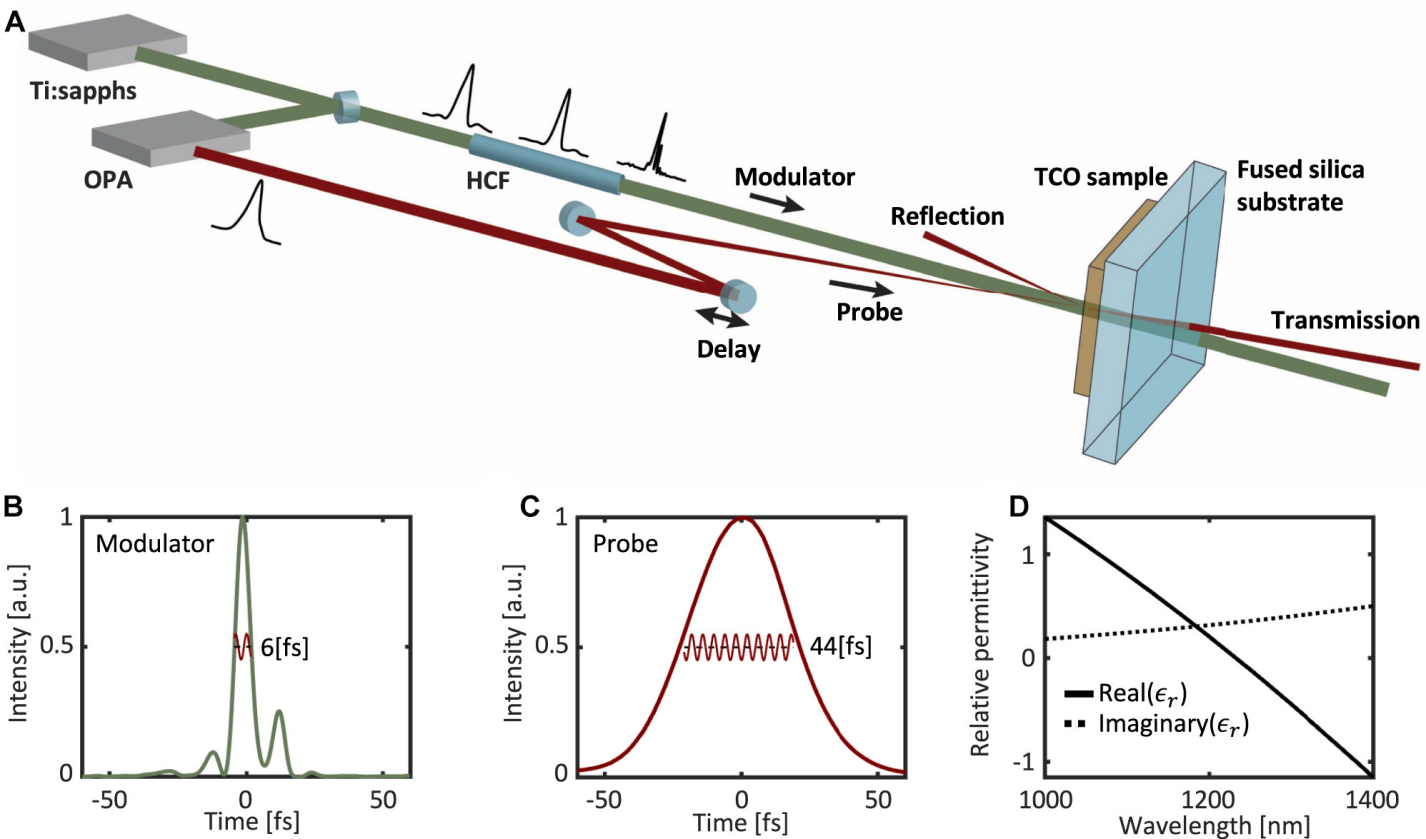
Experimental setup for measuring time-refraction in the single-cycle regime. (A) Schematics of the modulator – probe setup. The modulator is a pulsed optical beam at 800 nm (central) wavelength, compressed to different pulse durations through a hollow core fiber (HCF) system, illuminating a 700 μm diameter region of the sample. The probe beam is a down-converted 40 fs pulse at 1200 nm wavelength. The modulator and probe pulses are synchronized and arrive at different relative delays to the TCO sample. The intensity and spectrum of the transmitted probe and the intensity of the reflected probe are measured. (B) Intensity profile of a 6 fs FWHM modulator pulse (as retrieved through FROG), as generated by compression in the hollow core fiber. The red wiggly line marked in the main lobe illustrates the wave oscillations of the probe within the modulator pulse, showing that the probe pulse experiences less than two oscillations within the modulator pulse. (C) Intensity profile of the probe pulse, as retrieved by FROG. The red wiggly line marked in the main lobe illustrates the wave oscillations of the probe. The actual carrier envelope phase was not measured. (D) Real and imaginary parts of the permittivity of the 310 nm thick ITO sample as measured by ellipsometry.

To experiment with modulation of the refractive index at optical single-cycle rates, we need first to generate a modulator at these time durations. For this purpose, we use a hollow core fiber (HCF) filled with Ar gas to compress [41] a 30 fs 800 nm pulse down to only 6 fs. This pulse is used to modulate the index of refraction of a TCO material (Figure 1A). The medium we use is indium tin oxide (ITO), in the form of thin slabs of 0.31 μm thickness. Figure 1B shows the FROG (frequency resolved optical gating) reconstructed intensity profile of the maximally-compressed modulator pulse. To explore the single-cycle index modulation, we use a probe wave of a longer pulse (Figure 1C) in the NIR, with its central frequency near the ENZ point at 1225 nm (Figure 1D). Since the optical cycle of the NIR light is 4–5 fs, it is similar to the duration of the modulator pulse. Thus, for the probe beam – the modulation occurs within the time frame of a single temporal cycle (Figure 1B). The modulator beam illuminates a broad region in the sample, whereas the probe beam is focused in a much smaller region, such that the medium can act as virtually-homogeneous for the probe pulse in the transverse direction. The modulator power density on the sample is ∼**2** $\left[TW/cm^{2}\right]$ whereas the probe power density on the sample is five orders of magnitude smaller. We measure the intensity of the probe and its spectrum after it exits the material into free space, as well as the Fresnel reflection from the front interface of the ENZ sample.

## 4 Experimental results

**Figure 2:**
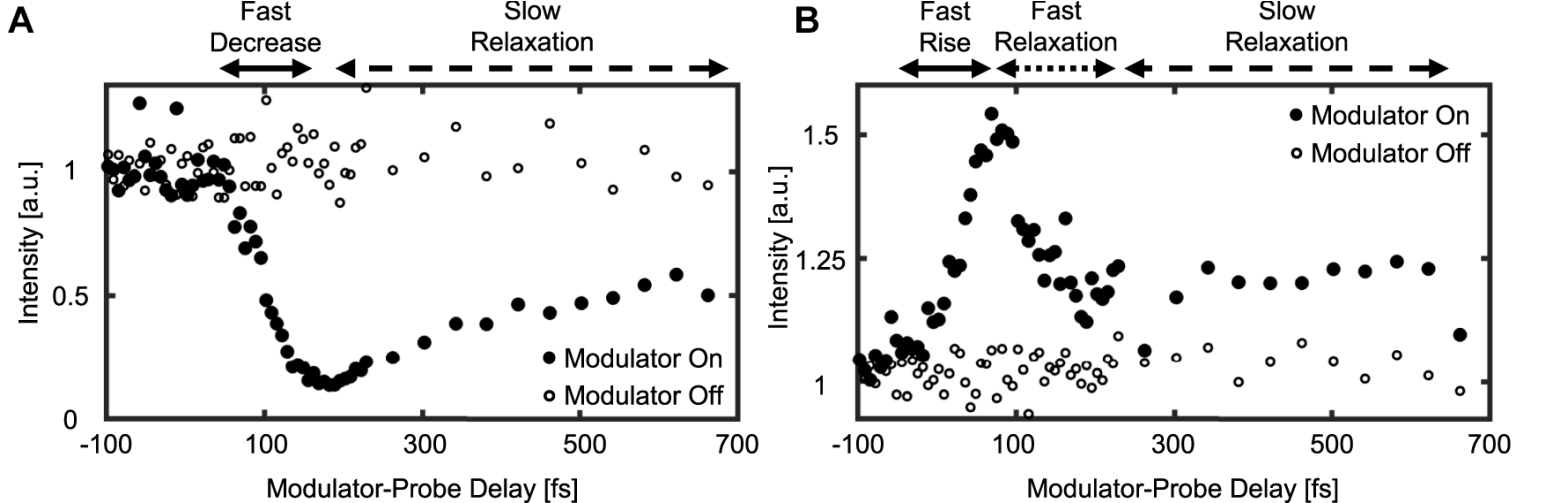
Effect of the ITO modulation (modulator FWHM = 6.8[fs]) on the Fresnel reflection and transmission. Negative delays (left side of each plot) correspond to the probe pulse passing through the sample before the modulator arrives. Zero delay is set to be the delay for which the probe pulse begins to be affected by the modulation. We estimate this point (on the data) by choosing zero delay as the point where 20 % rise of the transmitted probe is reached in (B). (A) Intensity of the Fresnel-reflected probe as measured by a photo-diode versus the delay between the modulator and the probe. Full circles correspond to measurements in which the ITO is modulated by the modulator, and hollow circles are reference measurements without modulation. (B) Intensity of the transmitted probe as measured by a photo-diode versus the delay between the modulator and the probe. Full circles correspond to measurements in which the ITO is modulated by the modulator, and hollow circles are reference measurements without modulation.

First, we examine the change in the intensity of the transmitted and Fresnel-reflected probe as a function of the modulator-probe delay, as measured on the output facet of the ITO sample (Figure 2). To do that, we image the transmitted and reflected output facets of the sample onto photo-diodes. We vary the delay between the two pulses and plot the temporal evolution of the reflected (Figure 2A) and transmitted (Figure 2B) intensity of the probe pulse, for each value of the delay. For reference, at each delay point we plot the transmitted and reflected intensity with the modulator pulse blocked. As Figure 2 shows, the transmitted and reflected intensity change dramatically by the modulation of the ITO. Examining the transmission and reflection as the probe and modulation start to overlap (leftmost part of Figure 2A and B), we see similar behavior to what was observed by other experiments with considerably longer modulation pulses (80–100 fs) [16, 33–37]. Mainly, the Fresnel reflected intensity decreases due to the modulation of the ITO and the transmission increases. As reported earlier, the relaxation of the Fresnel reflection takes several hundreds of femto-seconds. Surprisingly, the relaxation of the transmission shows a completely different picture. Namely, following the increase of the transmission as the refractive index rises in response to the modulation, we also see a sharp decrease of similar time scale as the refractive index decreases to a lower value and only after the fast partial relaxation – we also observe a slower relaxation that returns the index back to its original value. These observations are discussed below.

From the maximal transmission and reflection change, we can roughly estimate the index change amplitude caused by the modulation. By assuming, for simplicity, a plane wave and assessing the mean wavelength of the probe pulse, we can use the transfer matrix method and estimate (see details after Figure S6 in Section 1 of the Supplementary Information) that the real part of the refractive index is modulated by as much as ∼0.5 and the imaginary part is decreased by ∼0.15.

**Figure 3:**
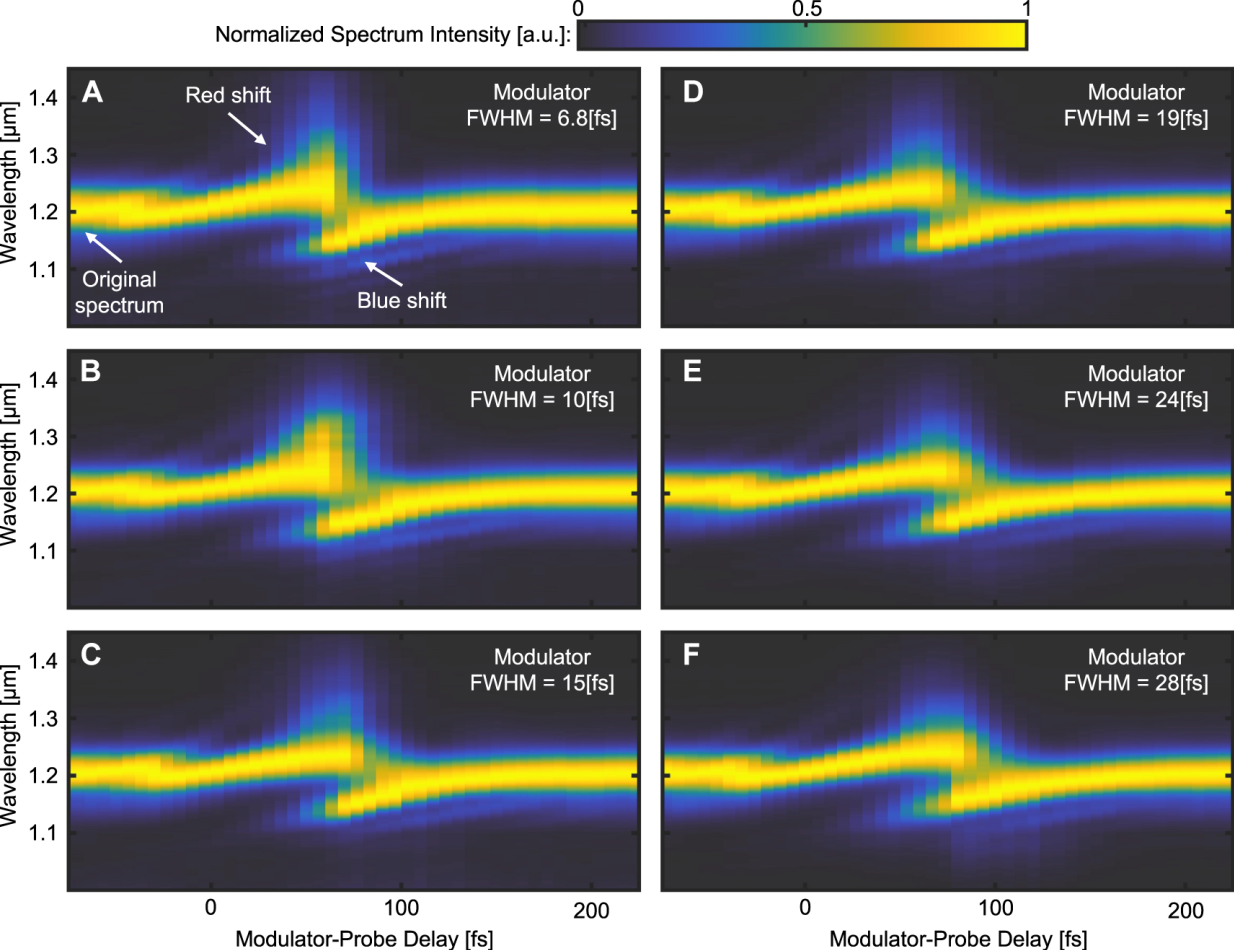
Transmission spectrograms of 44 fs probe pulses that have passed through the ITO sample, for modulator pulses of different temporal widths. For each panel, the zero delay time is arbitrarily set for a red-shift of 20 % of the maximal red-shift value in each experiment. (A–F) Spectrograms of the probe pulse transmitted through the sample, for modulator pulses of the temporal width (FWHM) listed at the top of each panel. Negative delays (left side of each plot) correspond to the probe pulse passing through the sample before the modulator arrives. Each spectrum of the spectrograms is normalized individually in each panel. Several features change between the panels as the modulator width shortens: the range of delay points in which red-shift is observed decreases as the temporal width of the modulator is decreased. Similarly, the range of delay points in which blue-shift is observed also decreases with the temporal width of the modulator. The maximal spectral red-shift increases as the modulator width is shortened.

Next, we examine the spectrum of the transmitted probe pulse as a function of the pulse duration of the modulator, as measured on the output facet of the ITO sample (Figure 3). To do that, we image the output facet of the sample onto a spectrometer. We vary the delay between the two pulses and plot the temporal evolution of the spectrum on a colormap, for each value of the delay. As Figure 3 shows, the spectrum of the transmitted probe pulse displays very large translation: first a red-shift as the index rises due to the modulator pulse, and subsequently a blue-shift as the refractive index relaxes back to its original value. This broad band non-resonant shift of the entire spectrum is expected to occur when a large index change (real or imaginary) is induced. However, the properties of this shift and how it evolves, especially down to the single-cycle regime, have never been studied. Each plot in Figure 3 shows a red-shift (increasing wavelength) and blue-shift (decreasing wavelength). The red-shift occurs when the probe experiences an increase in the refractive index which leads to decrease in its frequency, which we measure as an increase in the vacuum wavelength when light exits the sample. Likewise, the blue-shift occurs when the probe experiences a decrease in the refractive index. Interestingly, as shown in Figure 3, there is a region of delay values where the red-shift and blue-shift occur simultaneously for the same probe pulse. This region of simultaneous red and blue frequency shifts occurs because the probe pulse and refractive index response have similar durations, while the time it takes for the probe pulse to pass through the sample (on the order of 1 fs) is much shorter. Hence, in these experiments, the leading edge of the pulse experiences an increasing refractive index and is therefore red-shifted, whereas the trailing edge experiences only decreasing index and is blue-shifted. In addition, under this set of parameters, the center of the probe pulse – which experiences both increase and decrease of the refractive index – is negligible, and therefore the original peak of the spectrum disappears for these delay values.

Comparing the time scales of the changes to the transmission and Fresnel reflection in Figure 2 to the time scales of the frequency shifts in Figure 3 shows surprising results. The fast increase in transmission intensity (when the refractive index rises due to the modulator pulse) and the subsequent decrease in transmission intensity (relaxation), as measured by the photo-diode, align well with the red-shift and blue-shift of the transmitted spectrum (respectively). However, the Fresnel reflected probe acts differently. Namely, the fast decrease in the Fresnel reflection (when the index rises in response to the modulator) aligns well with the red-shift, **but** the increase in the reflected intensity (when the index relaxes) displays response time that is much longer then the time scale of blue-shift of the transmitted spectrum and of the relaxation of transmitted intensity.

## 5 Analysis

**Figure 4:**
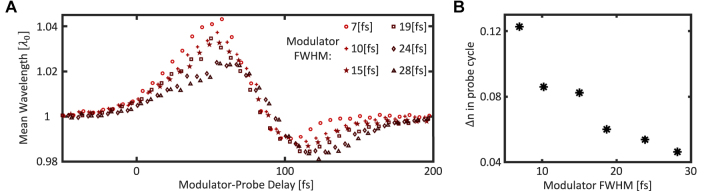
Analysis of transmitted spectrum as a function of temporal width of the modulator. (A) Mean wavelength (wavelength-weighted mean of the spectrum) of the transmitted probe pulse versus modulator-probe delay, for different modulator pulse widths. For each modulator width, the zero delay time is arbitrarily set for a red-shift of 20 % of the maximal red-shift value in each experiment. The rise time of the red-shift decreases for shorter modulator pulses and the red-shift become larger. (B) Maximal refractive index change occurring within a single cycle of the temporal period of the probe versus modulator width. Each data point is obtained by fitting the rise of the red-shift in Figure 3A to the error function convolved with the probe envelope, and using Eq. (2).

It is essential to analyze the transmitted pulse measurements of Figure 3 as a function of the modulator width. In Figure 4A, we plot the wavelength shifts of the mean wavelength (wavelength-weighted mean of the spectrum) versus modulator-probe delay, as extracted from the measurements displayed in Figure 3. These wavelength shifts show clear dependence on the modulator pulse width, both in the duration of the rise time and in the magnitude of the red-shift. The first observation that stands out is that shorter modulator pulses yield larger spectral red-shifts that occur within faster rise times. The faster rise times conform with the understanding that the refractive index change occurs because the material absorbs the modulator light, which excites conduction electrons to higher energies in the conduction band. Since absorption is an induced transition process, it is fundamentally instantaneous, hence it is expected that the rise of the refractive index would follow the change in the density of the excited (energetic) electrons, which is proportional to the accumulation of energy absorbed in the material. That is

(1) $$\frac{d}{dt}\Delta \varepsilon \left(t\right)\approx I_{\text{modulator}}\left(t\right)-\frac{1}{T_{\text{decay}}}\Delta \varepsilon \left(t\right)$$

where *I*_modulator_(*t*) is the time-dependent intensity of the modulator pulse (in units of [permittivity/time]) and *T*_decay_ is the relaxation time of the electrons back to their original state. From the solution of Eq. (1), we can calculate the temporal dependence of the index of refraction:

(2) $$n\left(t\right)=Re\left\{\sqrt{\varepsilon_{I_{\text{modulator}}=0}+\Delta \varepsilon \left(t\right)}\right\}$$

where $\varepsilon_{I_{\text{modulator}}=0}$ is the ambient (unmodulated) permittivity of the TCO slab. Since in our setup we probe the index change with a probe pulse, the refractive index response apparent in our measurements is a convolution of the index response and the temporal envelope of the probe pulse. In Figure 4B we present the refractive index change occurring within a single cycle of the temporal period of the probe, as calculated from the mean wavelength shift shown in Figure 4A and described by Eq. (2). For the shortest modulator width, we estimate that the refractive index of the sample is varied by ∼0.12 within a single cycle of the probe wave, and it takes 3–4 cycles (of the probe wave) for the refractive index change to rise to its maximal value of 0.5.

Examining Figures 3 and 4 leads to several observations. First, the probe pulse transmitted through the modulated sample experiences red-shift and blue-shift (and even overlap of red and blue shift). Second, the red-shift and blue-shift display similar durations, implying that their underlying effects have similar time scales. The time scales of both red-shift and blue-shift become shorter with a shorter modulator width. Finally, the maximal spectral shift of the mean wavelength increases as the modulator width is shortened, implying that a larger portion of the probe spectrum is red-shifted.

Importantly, we observe the same effects with several samples of the same material (ITO). The full data collected for the measurements presented in Figures 2 and 3 and measurements with other ITO samples are given in Section 1 of the Supplementary Information.

## 6 Modeling and simulations

**Figure 5:**
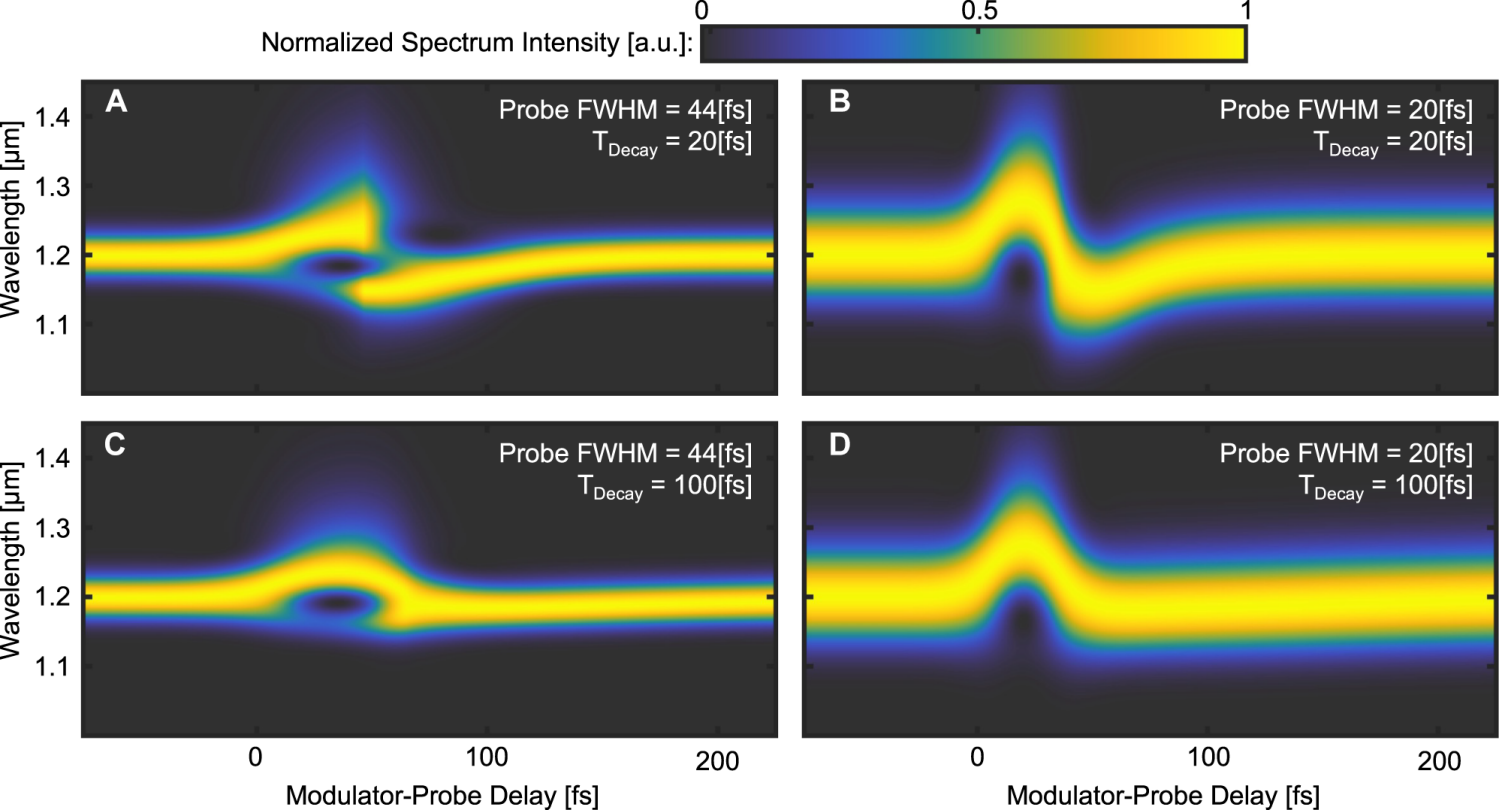
FDTD simulations of the transmission spectrograms of probe pulses passing through a slab whose refractive index is varied in time. The rise time of the refractive index (caused by a 20 fs FWHM Gaussian modulator pulse) and the slab width are the same in all simulations. For each panel, the zero delay time is arbitrarily set for a red-shift of 20 % of the maximal red-shift value in each simulation. The temporal width (FWHM) of the probe pulse and the relaxation time of the refractive index in each simulation are listed at the top of each panel. Negative delays (left side of each plot) correspond to the probe pulse passing through the slab before the slab refractive index starts changing. Each spectrum is normalized individually in each panel. (A) Spectrogram of a 44 fs FWHM probe pulse transmitted through the time-varying slab with a relaxation time of 20 fs. The results of this simulation resemble the experimental results to a high degree. (B) Spectrogram of a Gaussian 20 fs FWHM probe pulse transmitted through the time varying slab with a relaxation time of 20 fs. This simulation shows that the overlap between red-shift and blue-shift observed in Figure 2 arises from the long duration of the probe pulse. (C) Spectrogram of a 44 fs FWHM probe pulse transmitted through the time varying slab with a relaxation time of 100 fs. This simulation shows how the blue-shift depends on the relaxation time of the slab. Namely, when the relaxation is long (hundreds of femtoseconds), there is no blue-shift. (D) Spectrogram of a Gaussian 20 fs FWHM probe pulse transmitted through the time varying slab with a relaxation time of 100 fs. This simulation shows what can be expected from a slowly-decaying slab probed with a short probe pulse: the blue-shift observed in the experiments disappears when the relaxation time is 100 fs (irrespective of how short is the probe pulse).

To understand the mechanisms underlying these observations, we carry out simulations of a probe pulse refracting through a slab whose refractive index is varied in time at the relevant time scales. For these simulations, we assume that the slab changes its index following Eq. (2). For details on the simulations see, Section 2 of the Supplementary Information. The simulated transmission spectrogram, shown in Figure 5A, displays features similar to the experimental measurements shown in Figure 2. Figure 5B presents the simulation results of the same system, but with a shorter probe pulse of 20 fs FWHM. This simulated transmission spectrogram exhibits red-shift and blue-shift without any overlap between the shifts. From the results of this simulation, we deduce that the overlap in the red-shift and blue-shift, presented in Figure 3, is due to the long probe pulse we use, as mentioned above. Intuitively, this overlap occurs because the front of the probe pulse experiences the index rising and the back of the probe pulse experiences the index relaxing. Figure 5C shows the simulation results of the same system as in Figure 5A, but with a longer relaxation time of the refractive index. This simulated transmission spectrogram shows red-shift but almost no blue-shift at all. From the results of this simulation, we deduce that the relaxation time of the refractive index, as observed in our experiments, is on the scale of tens of femtoseconds, which is much faster than suggested in the previous studies with longer modulation pulses [15, 16, 33–37]. Those suggestions were based on the argument that the relaxation mechanism relies largely on phonons, which have relaxation times of hundreds of fs. Clearly, this is not the case in the experiments with single-cycle modulation. That is, as the simulation suggests, had the relaxation time been much longer than a few tens of fs, we would not have seen such large blue-shift. To complete the picture, Figure 5D shows the simulation results of a system consisting of a short probe pulse and a slow refractive index relaxation time. This simulation reaffirms that the fast relaxation time observed in our experiments and in the simulation of Figure 5A and B is due to the fast decay of the refractive index, and is not related to the probing wave. We can therefore say with high confidence that the relaxation time of 20–30 fs has a physical origin, and is not related to the measurement system.

## 7 Discussion

The dependence of the spectral red-shift on the width of the modulator pulse (Figure 4) calls for an explanation. There are several mechanisms that could contribute to that. The simplest explanation has to do with the finite thickness of the TCO sample. For an infinitely thick TCO sample, the frequency shifts would not depend on the rise time of the refractive index. On the other hand, when the TCO sample is thin enough so that the time it takes for the probe to pass through the sample is shorter than the rise time of the refractive index, the spectral red-shift depends on how much refractive index change the probe pulse experiences before leaving the sample. Intuitively, we can approximate the frequency shift to the ratio between the refractive index the wave experiences when it enters the sample and the refractive index it experiences when it leaves the sample. Under this approximation, it is clear that for thin samples (time of the probe to pass the sample < rise time of the index) the spectral red-shift will depend on the rise time of the index (which is related to the modulator width through Eq. (2)). This implies that, for long modulator pulses (long rise-time of the index) the probe will experience a smaller index change while traveling through the sample (and therefore smaller spectral red-shift) than with short modulator pulses. This intuitive explanation conforms to the red-shift of the mean wavelength observed in Figure 4A.

Another issue that merits discussion is the discrepancy between the relaxation times (of the refractive index) extracted from the intensity measurements in Figure 2A and B, which shows a difference in the relaxation times of the transmitted and Fresnel-reflected light. Specifically, the relaxation time experienced by the reflected light (hundreds of femtoseconds) is much longer then the relaxation time experienced by the transmitted light (10–20 fs). This fast relaxation time measured in the transmitted intensity agrees with the fast relaxation times of the spectral blue-shift, and therefore points towards slower relaxation at the surface, which affects the reflected light but not the transmitted light, such as surface plasmons created by the modulation. This said, the physical mechanism underlying the very fast relaxation time observed in our experiments is at this point unexplained. However, in ITO the fast relaxation phenomenon is apparent in all our experiments, with several samples of different widths (see Section 1 of the Supplementary Information). It is therefore a real and reproducible effect. It is important to emphasize that the whole process of temporal modulation of the refractive index is repeatable: the index rises and subsequently relaxes to the initial value, such that the material is under the same conditions when the subsequent modulator pulse arrives. This repeatable process of raising the refractive index within 1–2 optical cycles and subsequent relaxation within a few cycles give the hope that PTCs can be indeed generated at optical frequencies.

Before concluding, it is essential to add that we carried out similar experiments with another TCO material: AZO (aluminum zinc oxide). The experimental data, shown in Section 3 of the Supplementary Information, suggests that the rise time of the refractive index in AZO behaves in a way similar to ITO. On the other hand, the relaxation time in AZO is very slow and, as a result, almost no blue-shift is observed. In fact, the observed spectral shift in AZO resembles the simulations presented in Figure 5C and D, and is in agreement with what should be observed in the case of relatively slow decay of the refractive index. Thus, unlike the statement made in Ref. [42], what we show here, based on direct experimental observations, is that femtosecond time-varying effects in TCO are not obscured by other less exotic nonlinear effects (such as the optical Kerr effect and alike), but rather overshadow them.

## 8 Conclusions

To conclude, we studied experimentally a new regime in which a pulse propagates in a material with a refractive index that experiences very large variations (∼0.5) within a single-cycle time frame, while maintaining transparency. We analyzed and observed the behavior of the spectral shift as a function of the modulator width. Our measurements show that in ITO and AZO the refractive index can be raised within 5–10 fs to an index larger by up to 0.5 for ITO and 0.15 for AZO, and surprisingly, ITO can also exhibit extremely fast relaxation times of the refractive index – of 10–20 fs. Our findings, especially the very fast relaxation of the variation in the refractive index in ITO, and the fact that the process of raising the refractive index and subsequent relaxation to the original value, pave the way for observing photonic time crystals at optical frequencies, and many other phenomena involving time boundaries.

## Supplementary Material

Supplementary Material Details
