# Supplementary material for: Time-refraction optics with single cycle modulation
Source: Nanophotonics. 2023 May 31;12(12):2221–30. doi: 10.1515/nanoph-2023-0126 (PMC11501756; doi:10.1515/nanoph-2023-0126)
Supplement: Supplementary file 1 — Supplementary Material Details [file j_nanoph-2023-0126_suppl_001.docx]

**Time-refraction optics with single cycle modulation**

**Eran Lustig^1†^, Ohad Segal^1†^, Soham Saha***^2^***, Eliyahu Bordo^1^,** **Sarah N. Chowdhury^2^, Yonatan Sharabi^1^,** **Avner Fleischer^3^, Alexandra Boltasseva^2^, Oren Cohen^1^, Vladimir M. Shalaev^2^, Mordechai Segev^1^**

*^1^ Physics Department and Solid State Institute, Technion-Israel Institute of Technology, Haifa, Israel*

*^2^School of Electrical and Computer Engineering, Birck Nanotechnology Center and Purdue Quantum Science and Engineering Institute, Purdue University, West Lafayette, IN, USA*

*^3^ School of Chemistry, Tel Aviv University, Israel*

**^†^** *These authors contributed equally to this work*

[**msegev@technion.ac.il*](mailto:*msegev@technion.ac.il)

Supplementary Material

| **1. Experiments with ITO (Indium Tin Oxide)** |  |
| --- | --- |
| **2. Experiments with AZO (Aluminum Zinc Oxide)** |  |
| **3. Numerical simulation** |  |
| **4. References** |  |

In this Supplementary Material, we present additional experimental data that supports our observations and elaborate on the numerical simulations. In the first part (section 1), we present additional experimental data with the same Indium Tin Oxide (ITO) sample explored in the main text, as well as experiments with other ITO samples. In the second part (section 2), we present the data collected in an experiment with another TCO material: Aluminum Zinc Oxide (AZO). In the last part (section 3), we elaborate on the simulations presented in the main text.

**S.1 Experiments with ITO**

In this section we present additional data taken during the experimental measurements with ITO presented in the main text. First, we present, in Figs. S1-S6, the data collected in the measurements presented in Fig. 3 in the main text. Panels A in Figs. S1-S6 show the spectrum of the transmitted probe pulse for different modulator-probe delays. Panels B(C) in Figs. S1-S6 show the transmitted (reflected) pulse intensity for different modulator-probe delays. Each figure represents a set of experiments with a modulator pulse of a different temporal width.

In all of our experiments we measure, for different modulator delays, the spectrum of the transmitted probe (with a spectrometer), and the intensity of the transmitted probe and of the Fresnel-reflected probe, both with photodiodes.


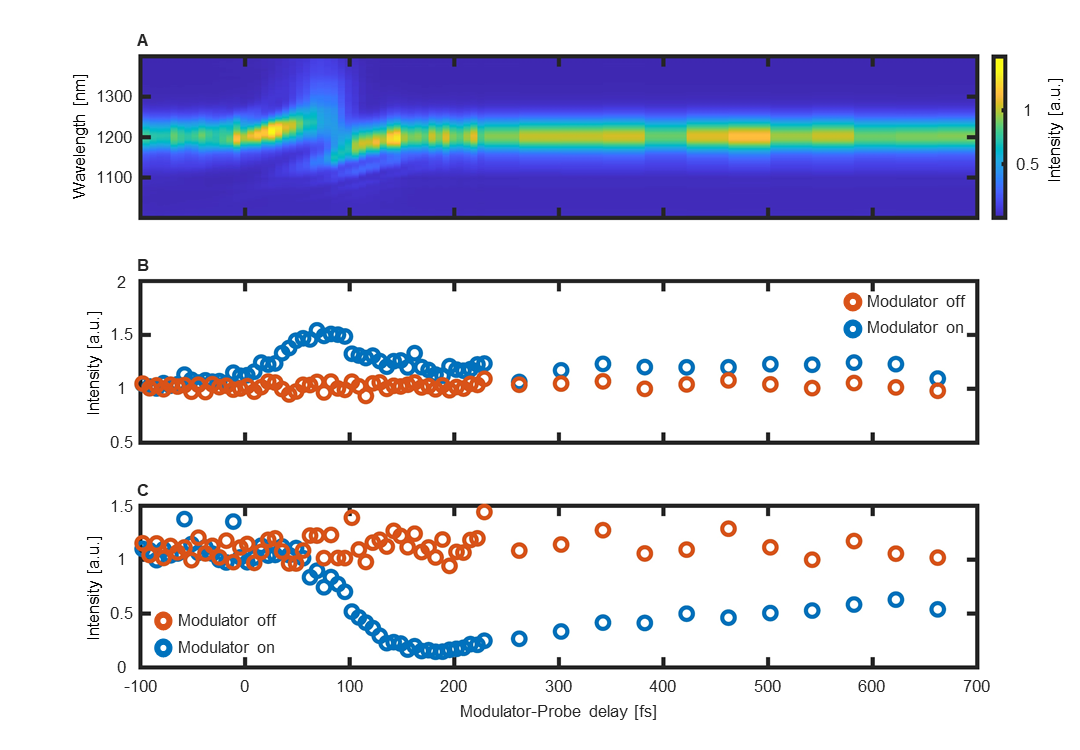


Figure S1: Transmission and reflection data of the modulator-probe experiment with modulator FWHM of 6.8[fs]. The zero delay time is arbitrarily set for a red-shift of 20% of the maximal red-shift value in A. (A) Spectrum of the transmitted probe pulse for different modulator-probe delays. (B) Intensity of the transmitted probe pulse measured by the transmission photodiode with and without the modulator pulse, for different modulator-probe delays. (C) Intensity of the Fresnel reflected probe pulse measured by the reflection photodiode with and without the modulator pulse, for different modulator-probe delays. Panel A is the same as Panel A in Fig. 3 of the main text, panels B and C are the same as panels B and A (respectively) in Fig. 2 of the main text.


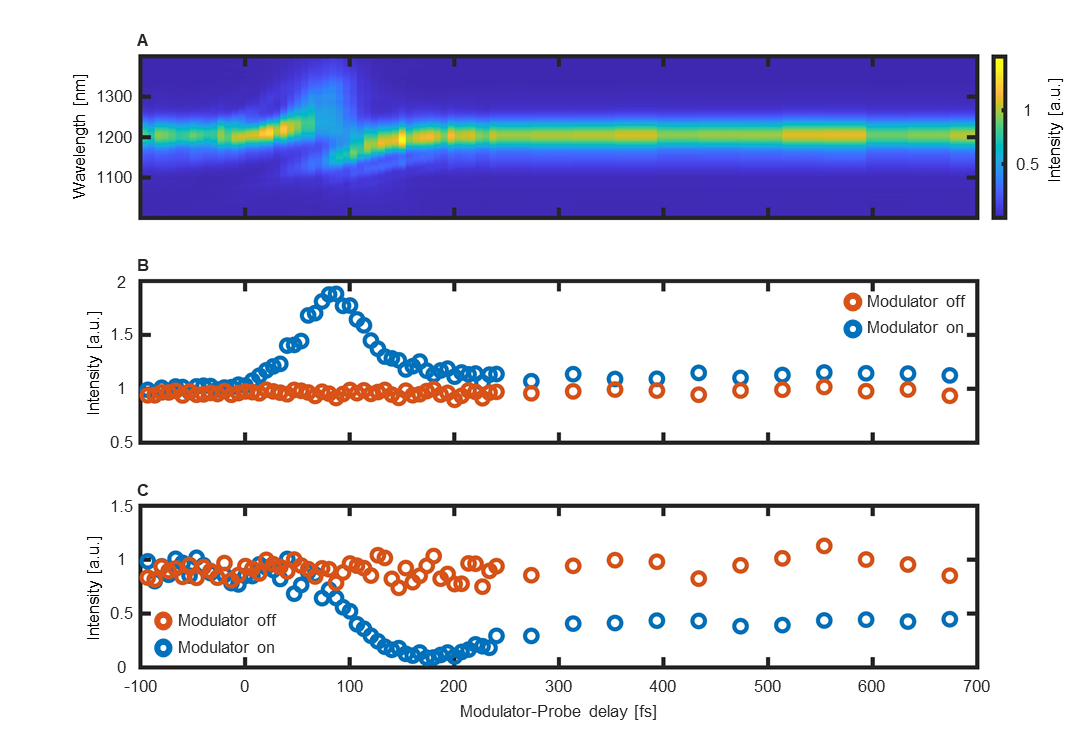


Figure S2: same as Fig.S1 only for modulator FWHM of 10[fs]. Panel A is the same as Fig. 3B of the main text.


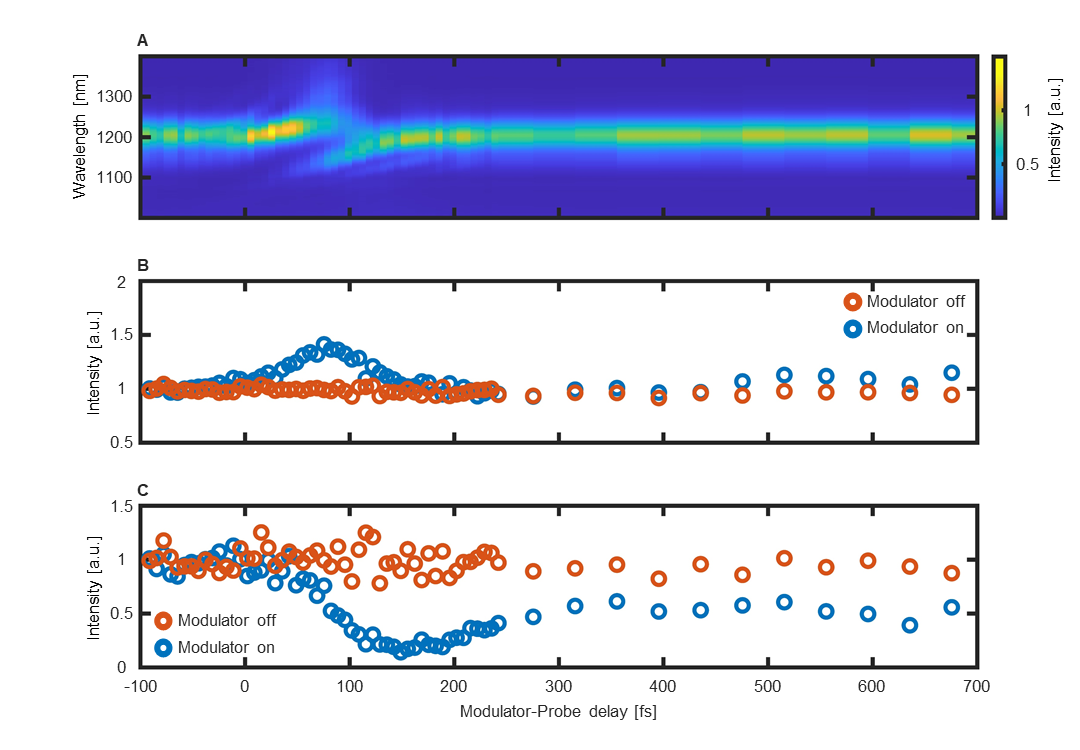


Figure S3: as Fig.S1-2 only for modulator FWHM of 15[fs]. Panel A is the same as Fig. 3C of the main text.


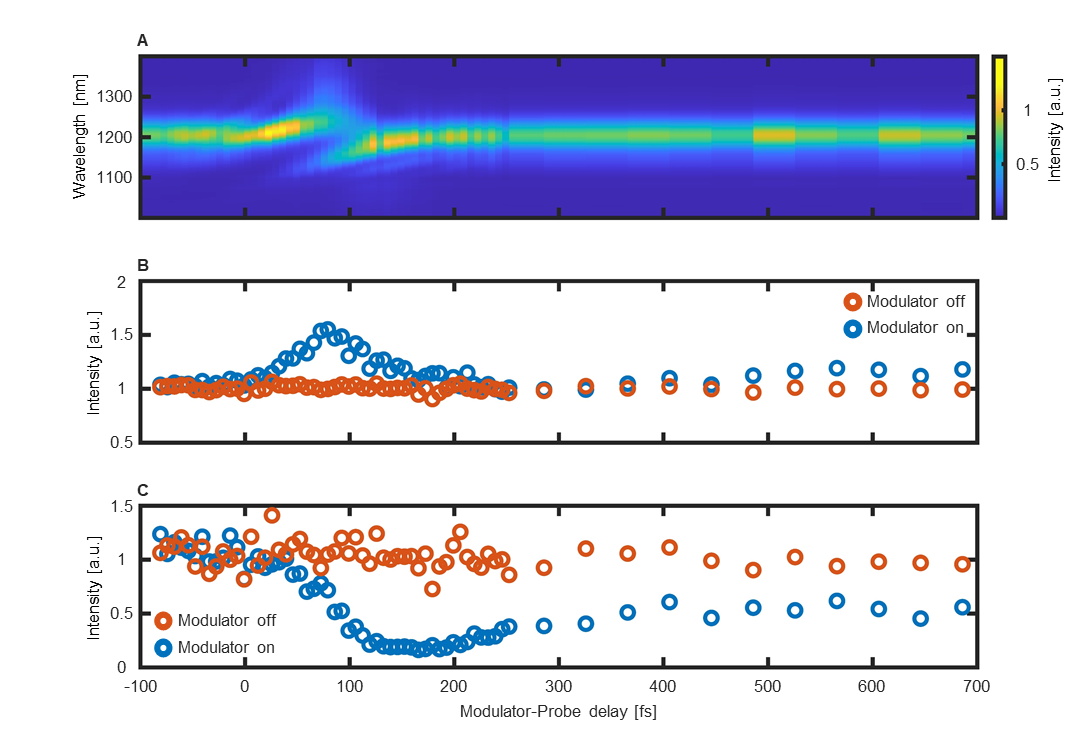


Figure S4: same as Fig.S1-3 only for modulator FWHM of 19[fs]. Panel A is the same as Fig. 3D of the main text.


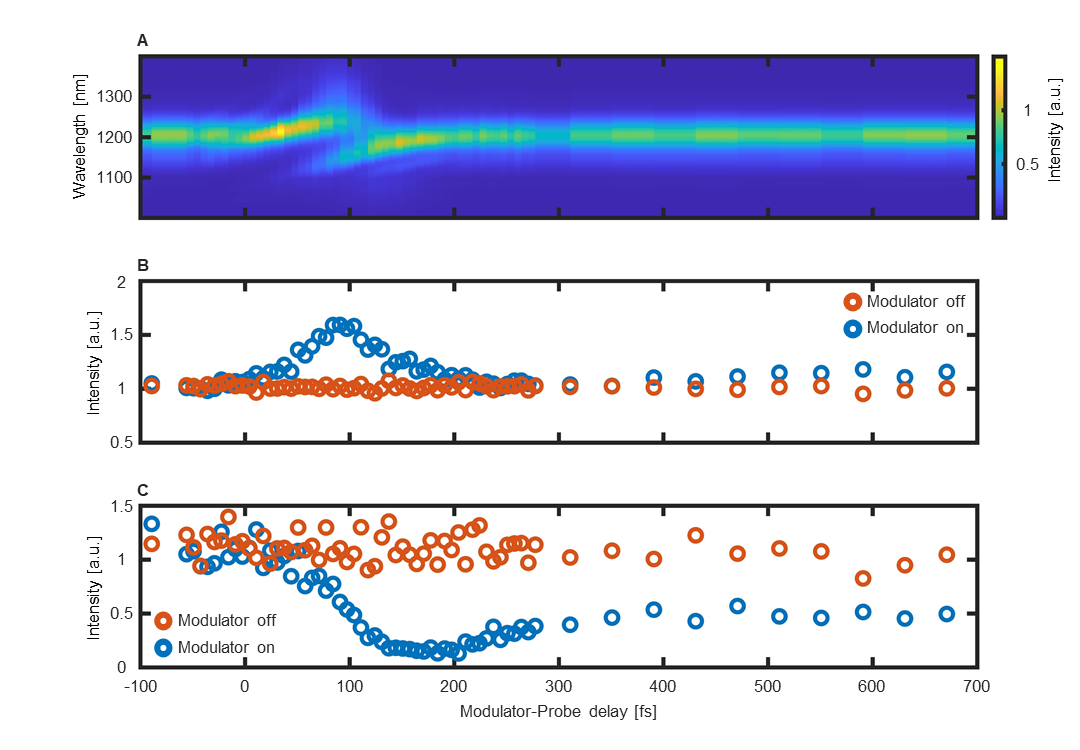


Figure S5: same as Fig.S1-4 only for modulator FWHM of 24[fs]. Panel A is the same as Fig. 3E of the main text.


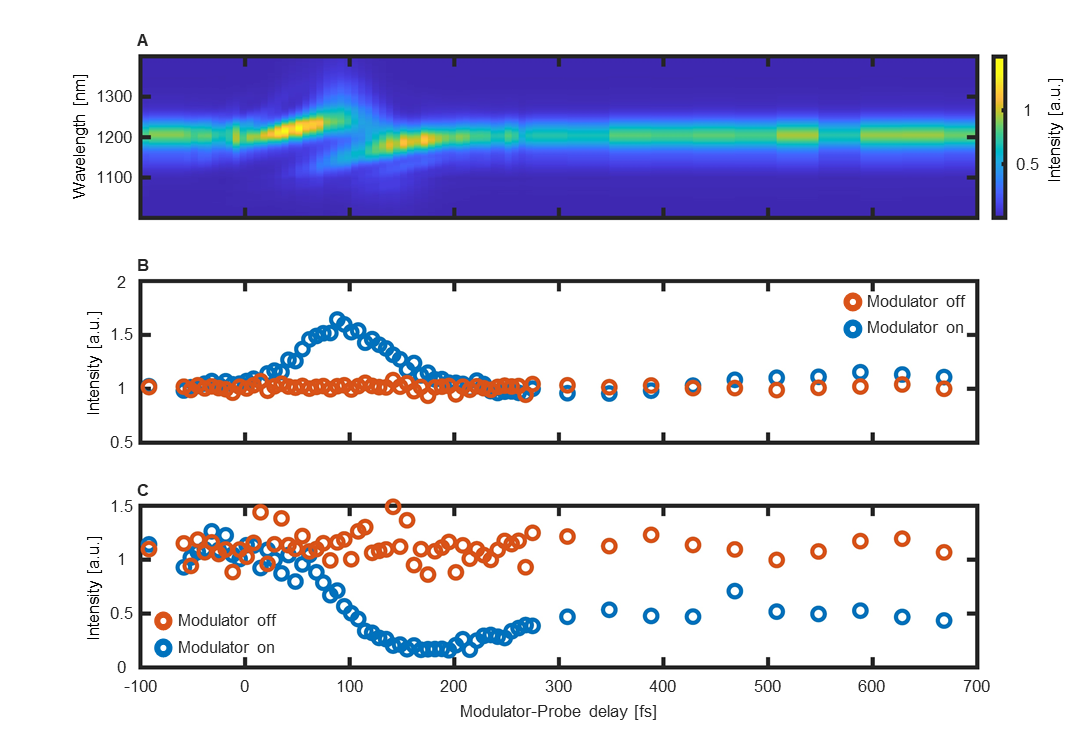


Figure S6: same as Fig.S1-5 only for modulator FWHM of 28[fs]. Panel A is the same as Fig. 3F of the main text.

Figures S1B,C-S6B,C show large modulations of the Fresnel coefficients. For each pulse duration of the modulator, we estimate the fully modulated real and imaginary refractive index using the maximally-transmitted and minimally-reflected values.

Since the incident beams are wide compared to the wavelength, we safely assume, for simplicity, that the index change is spatially-uniform and that the probe beam is a plane wave. We find the mean wavelength (wavelength-weighted mean of the spectrum) of the probe pulse, and use the Transfer Matrix Method (TMM) as follows. For a small angle of incidence (as in the experiment), we write the propagation matrix in layer $i$, $P_{i}$ and the transfer matrix from layer $i$ to $j$, $T_{i\to j}$:

|  | $P_{i}=\left( \begin{matrix} e^{-ik_{i}d_{i}} & 0 \\ 0 & e^{ik_{i}d_{i}} \end{matrix} \right), T_{i\to j}=\frac{1}{1-r_{i\to j}}\left( \begin{matrix} 1 & -r_{i\to j} \\ -r_{i\to j} & 1 \end{matrix} \right)$ | $\left( S1 \right)$ |
| --- | --- | --- |

where $i\in\left\{ air,ITO,Fused Silica \right\}$ is the layer, $k_{i}$ is the complex wavenumber in layer $i$, $d_{i}$ is the thickness of layer $i$ and $r_{i\to j}$ are the complex Fresnel reflection coefficients from layer $i$ to $j$.

Using these matrices, we write the total transfer matrix of our structure:

| $M_{tot}=T_{FS\to air}P_{FS}T_{ITO\to FS}P_{ITO}T_{air\to ITO}\equiv\left( \begin{matrix} A & B \\ C & D \end{matrix} \right)$ | $\left( S2 \right)$ |
| --- | --- |

And hence the intensity transmission and reflection coefficients are:

| $R_{tot}=\left\vert\frac{C}{D} \right\vert^{2}, T_{tot}=\left\vert\frac{1}{D} \right\vert^{2}$ | $\left( S3 \right)$ |
| --- | --- |

We use the values of the unmodulated refractive index (measured by ellipsometry) at the mean wavelength (Fig. 1D in the main text), the refractive index of the fused silica, and the thicknesses of the ITO and fused silica layers, to estimate the unmodulated transmitted and reflected intensity for the mean wavelength.

Next, we vary the real and imaginary parts of the refractive index of the ITO layer to fit the maximally-transmitted and minimally-reflected value we measure for each pulse duration of the modulator. That is, we find the real and imaginary part of the fully modulated ITO refractive index such that $\frac{R_{tot-Modulated}}{R_{tot-Unmodulated}}$ fits the minimal reflected probe intensity in Figs. S1C-6C and $\frac{T_{tot-Modulated}}{T_{tot-Unmodulated}}$ fits the maximal transmitted probe intensity in Figs. S1B-6B. Occasionally, this procedure yields several solutions. If more than one solution is obtained, we choose the one closest to the unmodulated value. Doing this with all pulse durations of the modulator, we deduce that in all these experiments the real part of the refractive index is modulated by ~0.5 and the imaginary part is decreased by ~0.15.

Given the experimental results presented in the figures of the main text and in Figs. S1-S6 here, it is essential to confirm that the effects we observe are indeed a general phenomenon and do not depend on the specific sample. To this end, we carry out a similar set of experiments with a thinner ITO sample which is 100nm thick (ENZ ~1235nm). The results are summarized in Fig. S7. The data taken with the thinner ITO sample shows the same features as in Figs. S1-6. Specifically, in Fig. S7A we observe the spectral red-shift, the blue-shift and the region where the red and blue shifts overlap, at similar delay times as in Figs. S1A-6A. The magnitude of the shifts in Fig. S7A is smaller than in Figs. S1A-6A, which is expected since the sample in Fig. S7 is thinner than the sample in Figs. S1-6 (as we explain after Fig. 5 in the main text). The relaxation time of the intensity of the transmitted and Fresnel-reflected probe pulse (Fig. S7B,C) is similar to what we see in Fig.2 in the main text and in Figs. S1B,C-6B,C.


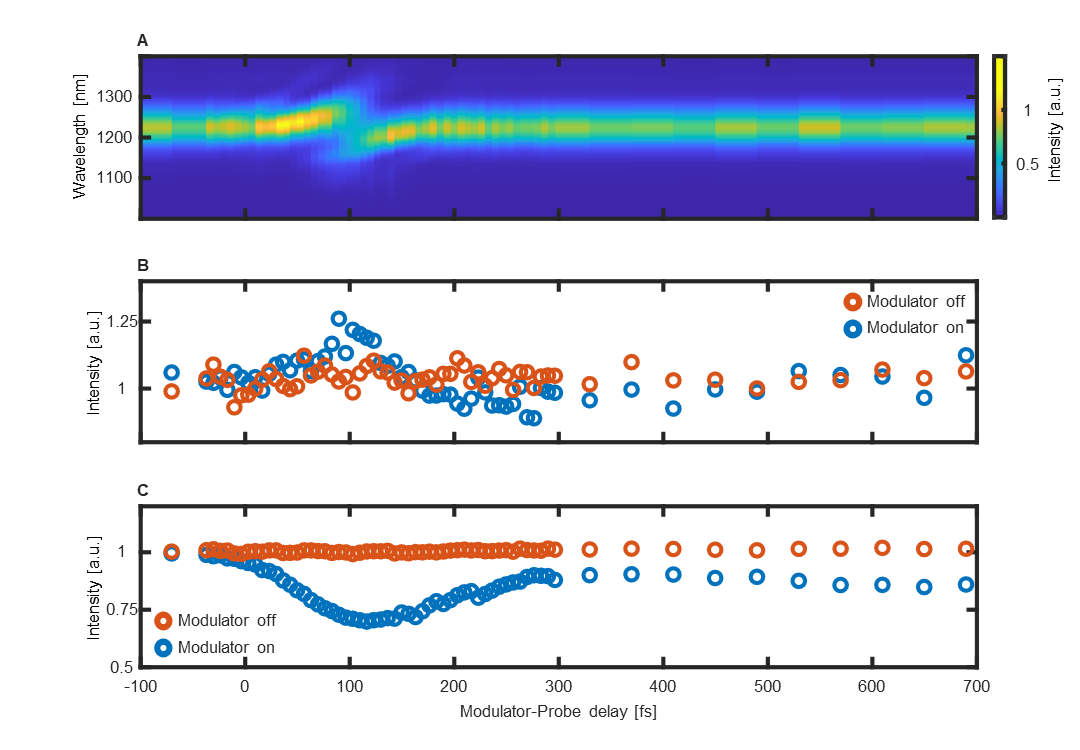


Figure S7: Transmission and reflection data of the modulator-probe experiment in a thinner sample of ITO (100nm thickness), and a modulator pulse of 28[fs] FWHM. The zero delay time is arbitrarily set for a red-shift of 20% of the maximal red-shift value in A. (A) Spectrum of the transmitted probe pulse for different modulator-probe delays. (B) Intensity of the transmitted probe pulse measured by the transmission photodiode with and without the modulator pulse, for different modulator-probe delays. (C) Intensity of the Fresnel reflected probe pulse measured by the reflection photodiode with and without the modulator pulse, for different modulator-probe delays.

Finally, we recall that Fig. 2 in the main text and Figs. S1-S7 show that the relaxation time of the transmitted and Fresnel-reflected probe intensity is different by more than an order of magnitude. One possible explanation for the difference could be surface effects which could in principle impact the Fresnel-reflected wave which is reflected from the surface, more than the transmitted wave. For example, the formation of a surface plasmon might in principle slow down the relaxation time of $n(t)$ at the surface, and possibly lead to the large difference between the fast relaxation times observed in both the spectral blue-shift of the transmitted probe and the transmitted intensity, and the much slower relaxation time of the Fresnel-reflected probe. To rule that out, we repeat our experiments with ITO with both probe and modulator entering the sample from the fused-silica substrate side (Fig. S8). Thus, if the modulator pulse gives rise to a surface effect, it would behave differently in the air-ITO interface and in the fused-silica – ITO interface. Figure S8 presents the data collected from a different ITO sample, of the same thickness as in the main text and in Figs. S1-6 (310 [nm]), but with both modulator and probe entering the ITO from the fused silica side of the sample. As Fig. S8 shows, there are no significant variations to the relaxation time scales of the transmitted and Fresnel-reflected probe intensity due to the change in the surface properties. While this measurement does not exclude completely surface effects, it does imply that the main effects we observe are not caused by surface effects.


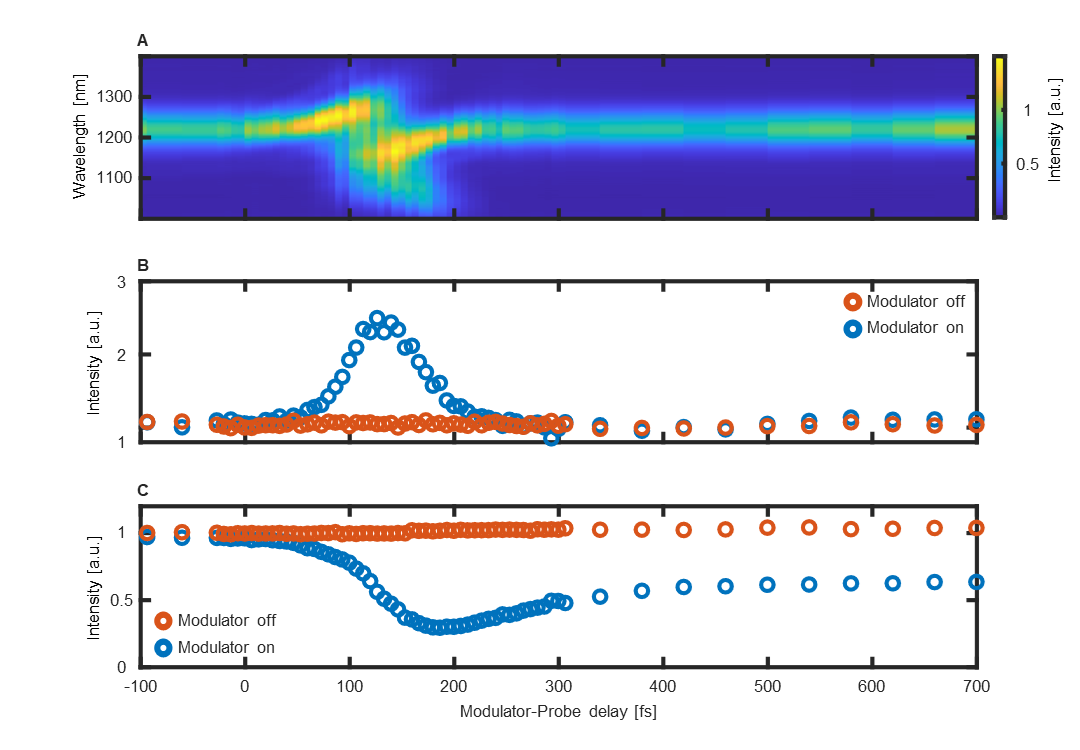


Figure S8: Transmission and reflection data of the modulator-probe experiment with modulator FWHM of 28[fs] and another 310[nm] thick ITO sample, where the modulator and probe both enter the sample from the fused silica-ITO interface (and not the air-ITO interface as in Figs S1-S7). The zero delay time is arbitrarily set for a red-shift of 20% of the maximal red-shift value in A. (A) Spectrum of the transmitted probe pulse for different modulator-probe delays. (B) Intensity of the transmitted probe pulse measured by the transmission photodiode with and without the modulator pulse, for different modulator-probe delays. (C) Intensity of the Fresnel reflected probe pulse measured by the reflection photodiode with and without the modulator pulse, for different modulator-probe delays.

**S.2 Numerical simulations**

To understand better the underlying mechanism behind the frequency shifts in our experiments, we preform Finite Difference Time Domain (FDTD) simulations of the Maxwell equations. As in the experiment, the simulations are carried out for a slab with refractive index that is modulated in time and a probe pulse that arrives with some relative delay with respect to the modulation. For simplicity, we neglect the dispersion and the losses of the TCO sample.

In our simulations, we examine a simple model using the 1D source-less Maxwell equations with dispersionless instantaneous permittivity ($D\left( t \right)=\varepsilon\left( t \right)E\left( t \right)$), and solve:

|  | $\begin{matrix} \frac{\partial E_{x}\left( t,z \right)}{\partial z}=-\mu_{0}\frac{\partial H_{y}\left( t,z \right)}{\partial t} \\ \frac{\partial H_{y}\left( t,z \right)}{\partial z}=-\varepsilon_{0}\frac{\partial\varepsilon_{r}\left( t,z \right)E_{x}\left( t,z \right)}{\partial t} \end{matrix}$ | $\left( S4 \right)$ |
| --- | --- | --- |

where $\varepsilon_{0}$ and $\mu_{0}$ are the vacuum permittivity and permeability and $\varepsilon_{r}\left( t,z \right)$ is the space- and time- dependent permittivity. To obtain the 1D equations from the general 3D macroscopic Maxwell equations, we set the initial conditions for the probe pulse as a pulsed plane-wave with electric field $\vec{E}=\left( E_{x},0,0 \right)$, magnetic field $\vec{H}=\left( 0,H_{y},0 \right),$ such that the only spatial dependence is along the z-direction.

We write these equations in a finite difference form using interlinked grid^1^.

|  | $\frac{E_{x}\left[ n,m+1 \right]-E_{x}\left[ n,m \right]}{\Delta z}=-\mu_{0}\frac{H_{y}\left[ n+\frac{1}{2},m+\frac{1}{2} \right]-H_{y}\left[ n-\frac{1}{2},m+\frac{1}{2} \right]}{\Delta t}$  $\frac{H_{y}\left[ n+\frac{1}{2},m+\frac{1}{2} \right]-H_{y}\left[ n+\frac{1}{2},m-\frac{1}{2} \right]}{\Delta z}=-\varepsilon_{0}\frac{\varepsilon_{r}\left[ n+1,m \right]E_{x}\left[ n+1,m \right]-\varepsilon_{r}\left[ n,m \right]E_{x}\left[ n,m \right]}{\Delta t}$ | $\left( S5 \right)$ |
| --- | --- | --- |

where $\Delta t$ and $\Delta z$ are the temporal and spatial sampling intervals of the temporal and spatial grids, respectively. In Eq. S5 we use square brackets notation to mark discretely sampled quantities (sampling intervals $\Delta t$ and $\Delta z)$ with $n,m\mathbb{\in Z}$ as the discrete time and space variables, respectively. As an example, the relation between the discretely sampled magnetic field and the continuous magnetic field is $H_{y}\left[ n+\frac{1}{2},m+\frac{1}{2} \right]=H_{y}\left( \left( n+\frac{1}{2} \right)\Delta t , \left( m+\frac{1}{2} \right)\Delta z \right)$. For stability and accuracy, we chose $\Delta t \left( \Delta z \right)$ to be at least an order of magnitude smaller than the smallest temporal (spatial) simulated detail and that $\Delta t<\frac{\min\left\{ \sqrt{\varepsilon_{r}} \right\}}{2}\Delta z$.

Equation S5 yields the following iterative update rules:

|  | $H_{y}\left[ n+\frac{1}{2},m+\frac{1}{2} \right]=-\frac{\Delta t}{\mu_{0}}\frac{E_{x}\left[ n,m+1 \right]-E_{x}\left[ n,m \right]}{\Delta z}+H_{y}\left[ n-\frac{1}{2},m+\frac{1}{2} \right]$  $E_{x}\left[ n+1,m \right]=-\frac{\Delta t}{\varepsilon_{0}\varepsilon_{r}\left[ n+1,m \right]}\frac{H_{y}\left[ n+\frac{1}{2},m+\frac{1}{2} \right]-H_{y}\left[ n+\frac{1}{2},m-\frac{1}{2} \right]}{\Delta z}+\frac{\varepsilon_{r}\left[ n,m \right]}{\varepsilon_{r}\left[ n+1,m \right]}E_{x}\left[ n,m \right]$ | $\left( S6 \right)$ |
| --- | --- | --- |

which we iterate over time to obtain the resulting fields given $\varepsilon_{r}\left[ n,m \right]$ and initial conditions.

The temporal structure of $\varepsilon_{r}\left( t,z \right)$ is assumed to be the solution of the ODE presented in the main text, Eq.1. The general solution of this ODE is a convolution between a decaying exponent with $T_{Decay}$ and $I_{modulator}$. For transform-limited Gaussian modulator pulse, the solution for$\Delta\varepsilon\left( t \right)$ is:

|  | $\Delta\varepsilon\left( t \right)=Ae^{-\frac{t}{T_{Decay}}}\left[ 1-\mathrm{erf} \left( \frac{\frac{\sigma_{modulator}^{2}}{T_{Decay}}-t}{\sqrt{2}\sigma_{modulator}} \right) \right]$ | $\left( S7 \right)$ |
| --- | --- | --- |

where $\sigma_{modulator}=\frac{FWHM_{modulator}}{2\sqrt{2\ln2}}$ is the temporal standard deviation of the gaussian modulator pulse, $T_{Decay}$ is the electrons relaxation time and $A$ is modulation amplitude.

To simulate our experimental system, we take

|  | $\varepsilon_{r}\left( t,z \right)=\left\{ \begin{matrix} \varepsilon_{TCO}+\Delta\varepsilon\left( t \right), & -\frac{D_{sample}}{2}\leq z\leq\frac{D_{sample}}{2} \\ 1, & else \end{matrix} \right.$ | $\left( S8 \right)$ |
| --- | --- | --- |

Where $D_{sample}$ is the sample thickness and $\varepsilon_{TCO}$ is the unmodulated permittivity of the TCO sample.

The simulation parameters are chosen such that the simulation highlights a heuristic model that exemplifies the experimental observations. To that end, we chose the parameters of the simulation to be similar to the actual parameters of our experimental system, while keeping in mind that some of our assumptions are too strong (especially: TCOs have strong dispersion and therefore slow group velocity near the ENZ point). Figure 5 show simulation results with $\varepsilon_{TCO}\mathfrak{=R}\left\{ n_{ITO}\left( 1200nm \right) \right\}^{2}=\left( 0.53 \right)^{2}$, which is a value we get from ellipsometry measurements of the ITO layer and appear in Fig. 1D in the main text. We chose the modulation amplitude $A$ such that the maximal change to refractive index will be 0.5, as we find from the maximal change in intensity of transmitted and Fresnel-reflected probe beam. We assume a transform-limited Gaussian-shaped modulator pulse with FWHM of 20fs and a transform-limited probe pulse with various temporal widths. In our experiment, the probe pulse has FWHM of 44[fs] but it is not a transform limited Gaussian. To simulate a probe with similar time-dependence as the probe in the experiment, we use a transform limited 44[fs] FWHM Gaussian. Last, we take the simulated optical thickness of the sample to be 4 times its actual thickness, in order to compensate for the low group velocity of the probe caused by the dispersion of the ITO (from the ellipsometry results we estimate that the group velocity of the probe pulse in the ITO sample is ~0.25 from the speed of light in vacuum).

We also include a video showing the response for gradually changing the duration of the probe pulse under fast refractive index decay time. This video shows how the spectrograms displaying the frequency shift develop between panels A and B in Fig. 5 in the main text. The video reaffirms that the duration of the probe pulse is indeed related to the splitting of the spectrum (simultaneous red and blue shift) apparent in our experimental results.

**S.3 Experiments with Aluminum Zinc Oxide (AZO)**

To gain better understanding of the time-refraction phenomena observed in our experimental system, we perform another set of experiments with another TCO material – aluminum zinc oxide (AZO). The sample we use is 1[um] thick and its ENZ point is ~1530[nm]. Figure S9 shows the data collected with AZO, 28[fs] FWHM modulator pulse, and a probe pulse of ~50fs FWHM and mean wavelength of ~1500[nm]. In these experiments, we observe a larger change in the transmitted intensity of the probe pulse (Fig. S9B), but a smaller change in the intensity of the Fresnel-reflected probe (Fig. S9C), as compared with the ITO experiments. Examining the time scales of the relaxation in both transmission and Fresnel-reflected intensity reveals that in this sample we do not observe a fast relaxation of the transmitted spectrum and intensity.


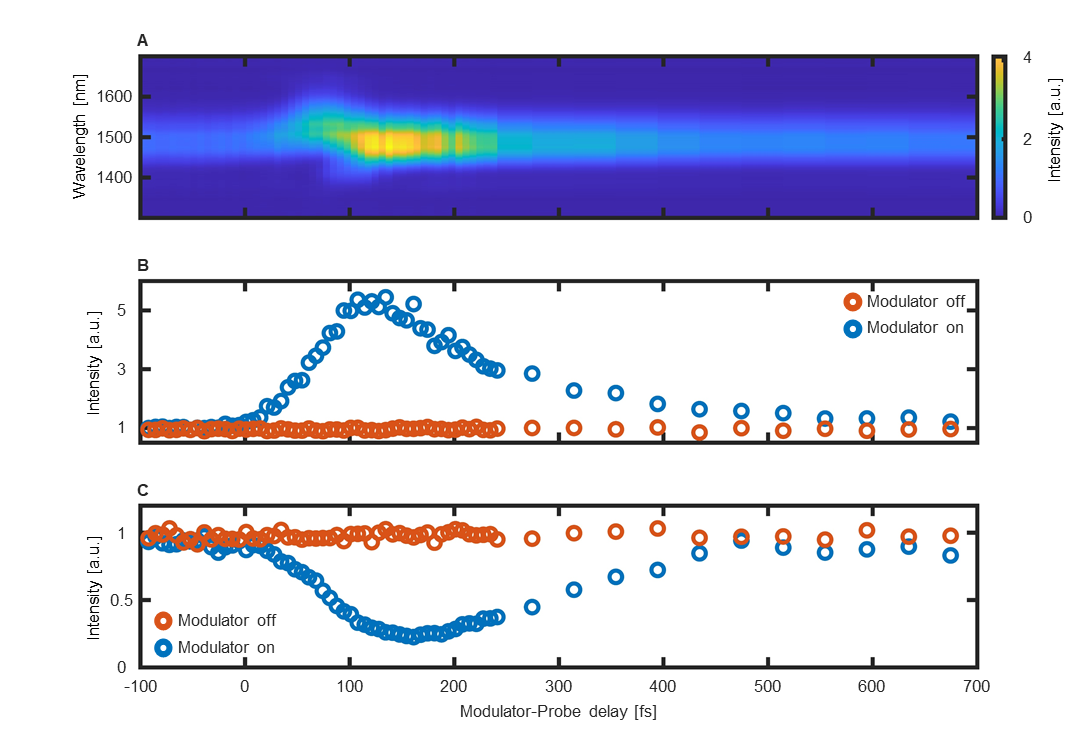


Figure S9: Transmission and reflection data of the modulator-probe experiment with modulator FWHM of 28[fs] and a sample of 1[um] thick AZO. The zero delay time is arbitrarily set for a red-shift of 20% of the maximal red-shift value in A. (A) Spectrum of the transmitted probe pulse for different modulator-probe delays. (B) Intensity of the transmitted probe pulse measured by the transmission photodiode with and without the modulator pulse, for different modulator-probe delays. (C) Intensity of the Fresnel-reflected probe pulse measured by the reflection photodiode with and without the modulator pulse, for different modulator-probe delays.

Figure S10 shows a spectrogram similar to the spectrograms in Fig. 3 in the main text. In this spectrogram each spectrum is normalized individually, so that frequency shift can be observed easily. Figure S10 shows a clear red-shift of the spectrum as the probe and the modulation start to overlap (left side of the plot), but, unlike all the experiments with ITO, with AZO no significant blue-shift is observed as n(t) relaxes.


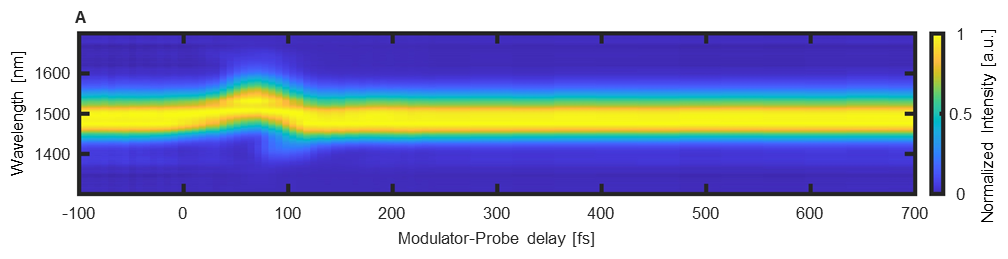


Figure S10: Transmission spectrogram of the probe pulse that has passed through the AZO sample, for modulator pulses of 28[fs] temporal width. The zero delay time is arbitrarily set for a red-shift of 20% of the maximal red-shift value. Negative delays (left side of the plot) corresponds to the probe pulse passing through the sample before the modulator arrives. Each spectrum of the spectrograms is normalized individually.

We note that, from the simulation results shown in Fig. 5C in the main text, this kind of behavior – of significant red-shift but virtually no blue-shift, is expected for slowly relaxing materials. From the combination of no spectral blue-shift and no fast relaxation of the change in the transmitted intensity of the probe pulse, we deduce that the refractive index in this AZO sample does not exhibit fast relaxation, as it does in ITO.

1. Yee, K. S. Numerical Solution of Initial Boundary Value Problems Involving Maxwell’s Equations in Isotropic Media. *IEEE Trans. Antennas Propag.* **14**, 302–307 (1966).
